# Endoplasmic reticulum homeostasis: a potential target for diabetic nephropathy

**DOI:** 10.3389/fendo.2023.1182848

**Published:** 2023-06-13

**Authors:** Ming Yang, Chongbin Liu, Na Jiang, Yan Liu, Shilu Luo, Chenrui Li, Hao Zhao, Yachun Han, Wei Chen, Li Li, Li Xiao, Lin Sun

**Affiliations:** ^1^ Department of Nephrology, The Second Xiangya Hospital, Central South University, Changsha, Hunan, China; ^2^ Hunan Key Laboratory of Kidney Disease and Blood Purification, Changsha, Hunan, China

**Keywords:** endoplasmic reticulum (ER), ER stress, UPR, ER-phagy, diabetic nephropathy

## Abstract

The endoplasmic reticulum (ER) is the most vigorous organelle in intracellular metabolism and is involved in physiological processes such as protein and lipid synthesis and calcium ion transport. Recently, the abnormal function of the ER has also been reported to be involved in the progression of kidney disease, especially in diabetic nephropathy (DN). Here, we reviewed the function of the ER and summarized the regulation of homeostasis through the UPR and ER-phagy. Then, we also reviewed the role of abnormal ER homeostasis in residential renal cells in DN. Finally, some ER stress activators and inhibitors were also summarized, and the possibility of maintaining ER homeostasis as a potential therapeutic target for DN was discussed.

## 1 Introduction

Diabetes is a serious disease that endangers human health, and long-term hyperglycemia can cause a variety of microvascular complications, including diabetic nephropathy (DN) (1). With the increasing number of patients with diabetes, DN has gradually become one of the main causes of end-stage renal disease (ESRD) (2, 3). However, symptomatic treatment is still the main treatment for diabetic nephropathy in clinical practice, and there is a lack of effective early diagnosis and treatment measures for DN. Therefore, in-depth elucidation of the molecular mechanism of DN is conducive to the development of DN treatment drugs.

The endoplasmic reticulum (ER) is one of the most metabolically active organelles involved in many cellular life processes. ER homeostasis is conducive to maintaining a relatively stable state of metabolic processes in cells, while ER dysfunction has also been shown to be closely related to the occurrence and development of a variety of diseases, such as liver disease (4), neurological diseases (5), diabetes (6) and cardiovascular disease (7). The role of ER in the pathogenesis of DN has also been partially revealed. In this review, we briefly describe the dysfunction of the ER in the intrinsic cells of the kidney in DN, and summarize some current stimulants and inhibitors of ER stress to maintain endoplasmic reticulum homeostasis as a potential therapeutic target for DN.

## 2 Function of the ER

In cells, the ER is classified as rough ER or smooth ER based on whether it is accompanied by ribosomes. The rough ER is mainly responsible for protein synthesis, while the smooth ER is the main site for lipid synthesis (8, 9). In fact, the most important and best-known functions of the ER is to participate in protein synthesis. In cells, the ER is required for more than one-third of protein synthesis, folding and structural maturation (10). Moreover, almost all proteins distributed in the ER, plasma membrane, Golgi apparatus, and lysosomes are translated on ER membrane-bound ribosomes (11). After the protein translation process is completed, different protein structures need to be formed through folding. In addition, the protein also needs to undergo posttranslational modifications, such as glycosylation, disulfide bond formation and oligomerization (12–14). These processes occur in the ER and are catalyzed by a large number of ER-resident enzymes, such as chaperones, glycosylases, and oxidoreductases (15, 16). When some proteins do not reach their native functional form once they are misfolded or improperly aggregated, these proteins need to be identified in a timely manner to avoid affecting cell function. In the ER, these misfolded proteins can be removed by the ER-associated degradation (ERAD) pathway to ensure cell function (17, 18). In the body, different tissues or cells have different abilities to synthesize and secrete proteins. For example, each beta cell of the pancreas can synthesize and secrete up to 1 million molecules of insulin per minute (19) and plasma cells can secrete their own body weight of antibodies every day (20). In general, cells with endocrine functions had a more active ER.

In addition to protein synthesis and folding, the ER is also involved in lipid synthesis. In fact, the ER is a core regulator of intracellular lipid levels. The ER can synthesize membrane lipids, including phosphatidylcholine (PtdCho) and phosphatidylethanolamine (PtdEtn). The ER is also rich in enzymes that synthesize cholesterol, as well as triglycerides (TAGs) for energy storage (21). Moreover, the ER is also involved in the process of lipid droplet formation (22, 23). In addition to its functions in protein and lipid synthesis, the ER is also essential for maintaining intracellular calcium homeostasis. The concentration of Ca^2+^ in the cytoplasm of cells is generally ~100 nM, while the concentration of Ca^2+^ in the lumen of the ER can reach 100-800 μM (24, 25). As a widespread signaling molecule in cells, Ca^2+^ affects a variety of biological processes including protein localization and function. When cytosolic Ca^2+^ levels are low, the ER maintains intracellular Ca^2+^ homeostasis by releasing Ca^2+^ through several Ca^2+^ channels, including ryanodine receptor and inositol 1,4,5-triphosphate receptor (IP3R) (26–28).

Thus, a properly functioning ER is essential for maintaining cellular homeostasis. However, ER dysfunction causes tissue and cell abnormalities and is closely related to the occurrence and development of a variety of diseases. The ER has corresponding self-regulation in response to changes in external stimuli.

## 3 Self-regulation of ER

### 3.1 The unfolded protein reaction

As mentioned earlier, the ER is involved in the proper folding process of proteins. However, under certain conditions of external stimulation, the cell is overloaded by protein synthesis. Thus, the workload of ER folding exceeds its capacity, and this state is known as ER stress (29). Persistent ER stress leads to the accumulation of misfolded proteins, which eventually leads to cell death (30–32). In response, cells have developed a system to monitor misfolded proteins at all times. When too much misfolded protein accumulates in the ER, the cell initiates a signal transduction pathway called the UPR in an attempt to correct the situation (17, 33). Briefly, UPR signaling is initiated by three ER transmembrane proteins: inositol-requiring enzyme 1α (IRE1α), pancreatic endoplasmic reticulum kinase (PERK), and activating transcription factor 6 (ATF6) (34). All of these proteins sense misfolded proteins directly or indirectly through an ER cavity domain, and the specific molecular mechanism was described in detail in our previous review (35). When there are too many misfolded proteins in the ER, they attempt to rebalance the need and capacity for protein folding through signaling pathways to maintain cellular homeostasis. UPR signaling expands the ER by increasing ER components (proteins and lipids) and upregulates the expression of chaperones to increase the protein folding capacity. The UPR also promotes the expression of certain genes to remove misfolded proteins in a timely manner. However, when ER stress is persistent, the UPR cannot reverse cellular homeostasis and will instead activate downstream molecules to promote cell death.

### 3.2 ER-phagy

Autophagy refers to the sequestration of misfolded proteins, damaged or aged organelles, and mutated proteins through the formation of autophagosomes, which eventually fuse into lysosomes to mediate the degradation of sequestered components (36). Macro-autophagy, micro-autophagy, and chaperone-mediated autophagy are three distinct forms of autophagy (37–39). Moreover, according to the different contents of degradation, macro-autophagy is also divided into mitophagy (40), ER-phagy (41), lipophagy (42) and so on. ER-phagy has been proposed as a novel way to regulate cellular ER homeostasis (41). At present, a variety of proteins (FAM134B, RTN3L, SEC62, CCPG1, ATL3 and TEX264) have been found to mediate ER-phagy. Their common feature is that they are located in the ER and can directly bind to LC3 through their LC3-interacting region (LIR) under specific conditions to mediate ER-phagy (35). Multiple cellular stresses, including starvation, the accumulation of misfolded proteins, and the imbalance of luminal calcium, can induce the occurrence of ER-phagy (35). In the presence of persistent ER stress, the UPR is overactivated and then activates the downstream proapoptotic signaling pathways, and the timely activation of ER-phagy can prevent the abnormal ER from further damaging the cells. The study of ER-phagy is still in its infancy, and the current view is that ER-phagy plays a protective role in ER quality control and that its effect is independent of the UPR pathway (41).

## 4 Abnormal ER function in DN

**Figure 1:**
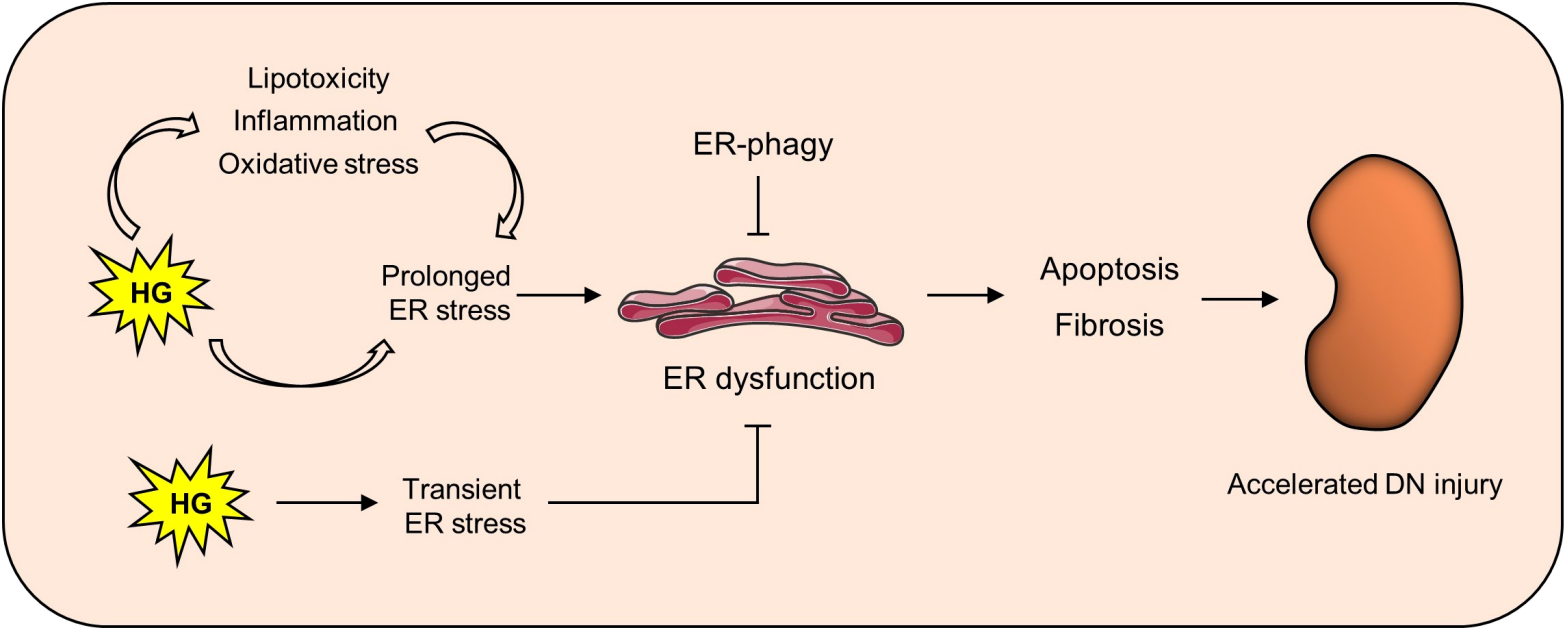
Maintaining ER homeostasis delays kidney damage in DN. In the diabetic state, high glucose can not only directly induce ER stress, but also activate it through lipotoxicity, oxidative stress and inflammation. Persistent ER stress can lead to ER dysfunction, thus causing downstream apoptosis and fibrosis and finally aggravating renal injury in DN.

The role of ER homeostasis in the occurrence and development of DN has attracted increasing attention. A surprising study has shown that in humans, the fractional rate of renal protein synthesis is estimated to be approximately 42% of the total daily load, which is the highest of any organ. This implies that renal cells may be highly sensitive to changes in ER homeostasis (43). With the deepening of the research on the pathogenesis of DN, abnormalities in ER homeostasis accelerate the progression of DN through different pathways (**Figure 1**), and some compounds or drugs may delay the progression of DN by maintaining ER homeostasis. Here, we will review in detail the indispensable role of ER homeostasis in DN progression.

### 4.1 ER stress

A number of studies have reported that there is excessive activation of ER stress in the kidney in DN. In the state of diabetes, the body engages a long-term chronic stress response process. At this time, ER stress is overactivated, which impairs the normal function of the kidney. Multiple factors have been reported to be involved in ER stress in DN. It was reported that prolonged exposure to high glucose directly activates IRE1, a key molecule of ER stress, thereby promoting the occurrence of ER stress (44). Moreover, advanced glycation end products (AGEs) are one of the typical pathogenic factors of DN (45, 46), and studies have shown that AGEs are also involved in the occurrence of ER stress in DN. Mechanistically, AGEs activate nicotinamide adenine dinucleotide phosphate (NADPH) oxidase (Nox) through receptor for AGEs (RAGE), leading to ROS generation, which subsequently leads to oxidative stress, ER stress and UPR accumulation (47). In addition, angiotensin II receptor, free fatty acids (FFAs) and oxidative stress have also been shown to be involved in the pathogenesis of ER stress in DN (48). Moreover, in the kidney, different cells have different responses to ER stress.

#### 4.1.1 ER stress in proximal tubular cells

Pathologically, the main manifestations of DN are glomerular sclerosis, tubular atrophy and renal interstitial fibrosis. It has been previously believed that glomerular damage is the basis of DN. However, with the in-depth study of the pathogenesis of DN, renal tubular damage seems to occur preferentially to glomerular damage (49). Bader et al. reported that there is a certain correlation between the vascular index and relative cortical interstitial volume and serum creatinine, and if there is little renal interstitial fibrosis, even severe glomerular pathological changes can manifest as normal creatinine levels. Conversely, patients with severe interstitial fibrosis, even mild glomerular changes, often have elevated serum creatinine levels (50). The main function of renal tubular cells is to reabsorb glucose and protein in urine. In diabetes, the ultrafiltration of the kidney causes the renal tubular cells to be overload (49). In turn, the ER of proximal tubular epithelial cells needs to synthesize more proteins to maintain renal function. Several studies have reported the activation of ER stress in tubular cells in DN.

Jiang et al. demonstrated enhanced ER stress, impaired autophagy and mitochondrial dysfunction in the kidneys of db/db mice (a commonly used mouse model of DN), accompanied by the upregulation of soluble epoxide hydrolase (sEH) expression. The specific inhibition of sEH expression by t-AUCB can significantly inhibit the level of ER stress and kidney injury in DN (51). Moreover, in cultured mouse proximal tubular epithelial cells, AGEs could upregulate the expression of RAGE, GRP78 and p21 in a dose- and time-dependent manner, accompanied by premature aging in DN. Further studies showed that AGE-induced premature aging could be mimicked by using an ER stress inducer or RAGE overexpression. However, this was significantly inhibited by p21 gene silencing, and inhibiting RAGE attenuated high glucose-induced ER stress and premature aging (52). In addition, dapagliflozin is a new type 2 diabetes drug that can reduce blood glucose levels and body weight by inhibiting sodium-glucose transporter 2 (SGLT2) in proximal tubular cells (53, 54). Moreover, it has also been shown to have renoprotective effects. Shibusawa et al. reported that it reduces the reabsorption of glucose into renal tubular cells, thereby regulating metabolic conditions that cause cellular ER stress. Mechanistically, it plays an anti-ER stress role by regulating the elf2α-ATF4-CHOP pathway (55). In addition, our previous study also showed the decreased expression of DsbA-L and the activation of ER stress in renal tubular cells of mice with STZ-induced DN. However, the overexpression of DsbA-L inhibited ER stress, and knockout of DsbA-L aggravated ER stress (56). These studies suggest that ER stress is overactivated in tubular cells of DN and that inhibition of ER stress can alleviate tubular injury in DN.

#### 4.1.2 ER stress in podocytes

Abnormalities in the glomerular filtration barrier maintained by podocytes are also involved in the progression of DN. In patients with diabetes, podocytes are susceptible to apoptosis by stimulation, and the massive loss of podocytes leads to the pathological development of proteinuria, mesangial expansion, and glomerulosclerosis (57–59). High glucose levels can further increase ER stress, podocyte phenotype switching and podocyte loss in rat kidneys, and these adverse effects can be inhibited by exogenous ER molecular chaperones (60). In addition, overactivated ER stress induces podocyte apoptosis. In podocytes treated with high glucose, the expression of ER stress markers, such as GRP78, was significantly increased, and the apoptosis of podocytes was induced (61). Recently, RTN1A-mediated ER stress has also been found to be involved in podocyte injury in DN. RTN1 belongs to the RTN protein family and was originally described as an ER-forming protein in neuroendocrine cells, where it mainly localizes to the ER membrane (62, 63). Subsequent studies found that only RTN1A expression was increased among three RTN1 isoforms (RTN1A, RTN1B, and RTN1C) and was associated with ER stress in human and mouse models of kidney disease (64). Fan et al. demonstrated that in podocytes, high glucose levels significantly upregulate the expression of RTN1A and ER stress levels. In addition, the overexpression of RTN1A in podocytes can also cause ER stress, while the inhibition of RTN1A expression notably attenuated the ER stress induced by high glucose (65). Further studies revealed that the N-terminal and C-terminal domains of RTN1A interact with PERK, which may be critical for its induction of ER stress (65). In general, ER-induced podocyte injury is critical for DN. However, the stimuli and mechanisms of ER stress in podocytes in the state of DN are largely unknown and require further investigation.

#### 4.1.3 ER stress in mesangial cells

Mesangial cells are important cells of the glomerulus, and their dysfunction is also involved in the occurrence and development of DN. Fatty acid binding protein 4 (FABP4) is a carrier protein of fatty acids (66). In the kidney, FABP4 was mainly expressed in glomerular mesangial cells in renal biopsy tissues, and its expression was significantly upregulated in renal biopsy tissues of patients with DN compared with control patients (67). A similar result was also observed that the expression of FABP4 is increased in mesangial cells treated with high glucose, accompanied by the upregulation of ER stress and apoptosis (67). However, treatment with the FABP4 inhibitor BMS309403 or siRNA reversed the adverse changes in ER stress and apoptosis caused by high glucose (67). This implies that FABP4-mediated ER stress is involved in the pathogenesis of DN. Moreover, the inhibition of ER stress by some compounds can alleviate mesangial cell injury in DN. Zhang et al. showed that ER stress and apoptosis were upregulated in the kidneys of DN mice and high glucose-treated mesangial cells, while thalidomide treatment notably inhibited ER stress and apoptosis (68).

In general, the presence of ER stress may have a certain protective effect in the early stage of the disease, which is beneficial for the timely cells to detection and adjustment of the abnormal intracellular protein synthesis in cells. However, as a chronic metabolic disease, long-term ER stress can aggravate cell damage and eventually lead to cell apoptosis, thereby accelerating the progression of DN. Therefore, the search for ER stress specific inhibitors may be a potential direction for drug development in DN.

### 4.2 ER calcium homeostasis and DN

The SERCA family mediates the uptake of Ca^2+^ from the cytoplasm by the ER, which drives Ca^2+^ across the membrane resistant to electrochemical gradients by consuming large amounts of ATP (69, 70). Guo et al. demonstrated that SERCA2 activity and expression were significantly reduced in the renal cortex of db/db mice, which led to Ca^2+^ depletion in the ER and ultimately induced ER stress and mitochondria-mediated apoptosis (71). Furthermore, treatment with astragaloside IV dose-dependently upregulated the expression and activity of SERCA2, restored intracellular Ca^2+^ homeostasis, and alleviated ER stress and cell apoptosis in podocytes stimulated with palmitic acid (71). Apart from Ca^2+^ homeostasis, the ER can also affect other organelle functions by controlling Ca^2+^ transport. The newly discovered mitochondria-associated ER membrane (MAM) is a subdomain of the ER (72, 73). MAMs are composed of parts of the ER, adjacent outer mitochondrial membranes, and proteins. One of its major functions is to mediate calcium transport from the ER to mitochondria (74, 75). In the presence of certain stimuli, the ER mediates calcium transfer from mitochondria through the IP3R/VDAC1 protein channel. An appropriate increase in mitochondrial Ca^2+^ concentration will increase ATP synthesis, thus making it more powerful to resist external adverse factors. However, when the calcium concentration in mitochondria exceeds a certain level, it will lead to mitochondrial calcium overload and eventually cause mitochondria-mediated apoptosis (76, 77). Our previous study showed that disrupted MAM integrity was observed in renal biopsy tissues from patients with DN, STZ-induced DN mice and HK-2 cells treated with high glucose, and restoring MAM integrity was beneficial in protecting against apoptosis induced by high glucose (56). Although increasing attention has been given to the role of ER-mediated calcium homeostasis in metabolic diseases, the molecular mechanism of ER-mediated calcium homeostasis and its role in the progression of DN still need to be further studied.

### 4.3 ER-phagy and DN

ER-phagy, as a selective autophagy, can remove ER stress in time to prevent further cell damage. During ER stress, misfolded proteins accumulate in the ER, most of which can be degraded through the ERAD pathway, but some that are not in the ERAD pathway can be degraded through the ER-phagy pathway. A fraction of misfolded peptides that fail the ERAD pathway are sequestered into dedicated ER subdomains by ER-resident chaperones, and they engage specialized ER autophagy receptors. Therefore, vesiculation of this portion of the ER and its subsequent lysosomal degradation are promoted (78). Previous studies have shown that when the mouse liver lacks the ER-phagy receptor FAM134B-2, a large amount of abnormally secreted proteins accumulate in mouse hepatocytes (79). Aberrant ER-phagy has been shown to be involved in the pathogenesis of nervous system diseases, tumors and infectious diseases (35), but unfortunately, the study of ER-phagy in kidney diseases has not been carried out. However, given the importance of ER-phagy in the maintenance of ER homeostasis, its role in DN needs to be further explored in future studies.

## 5 ER stress inhibitor

Reducing ER stress and maintaining ER homeostasis is a direction of targeted ER therapy for DN. At present, some compounds have been identified to inhibit ER stress and slow the progression of DN.

### 5.1 TUDCA

Taurine deoxycholic acid (TUDCA) is a naturally occurring hydrophilic bile acid that is the taurine conjugate of ursodeoxycholic acid (UDCA). It has been approved by the Food and Drug Administration (FDA) for the treatment of primary biliary cholangitis (80). In-depth studies have shown that it also plays a protective role in diseases such as diabetes (81), obesity (82) and cardiovascular disease (83). In terms of molecular mechanisms, it acts as a chaperone to inhibit ER stress. TUDCA has also shown protective effects in a DN model. Zhang et al. demonstrated that the blood glucose, proteinuria, renal pathological damage, ER stress and apoptosis levels were lower in intraperitoneal TUDAC-treated db/db mice than in control mice (84). Similar results were also observed that in ERp44-deficient db/db mice, which exhibited more severe ER stress, glomerular basement membrane thickening and proteinuria, and ERp44-depleted DN symptoms were ameliorated by TUDCA treatment (85). Moreover, Chen et al. found that TUDCA inhibited the AGE-induced expression of glucose regulated protein 78 (GRP78, an ER stress marker protein) and inhibited apoptosis in a dose-dependent manner in podocytes of DN mice (86). The molecular mechanism by which TUDCA alleviates ER stress is still unclear. As a molecular chaperone, TUDCA may alleviate ER stress by stabilizing misfolded proteins, stimulating chaperones to transport proteins more efficiently, and reducing protein aggregation (80).

### 5.2 4-phenylbutyric acid

In addition to TUDCA, 4-PBA is another commonly used ER stress inhibitor. It is an orally bioavailable low molecular weight fatty acid that has been clinically approved by the FDA for the treatment of urea cycle disorders and hyperammonemia (87). The molecular mechanism by which it inhibits ER stress is that its hydrophobic domain interacts with the exposed hydrophobic fragments of unfolded proteins, which promotes protein folding and prevents protein aggregation (88). The study showed that compared with control mice, db/db mice treated with 4-PBA showed less proteinuria and reduced glomerular mesangial expansion. Moreover, 4-PBA inhibited the expression of ER stress-related proteins, such as BiP, phospho-IRE1α, phospho-eIF2α and CHOP, and reduced renal cell apoptosis (61). A similar result was also observed in which 4-PBA alleviated ER stress, renal inflammation and renal injury in rats (89).

### 5.3 Aliskiren

Aliskiren is the first direct renin inhibitor that is effective orally. In the DN state, the activation of the renin system is an important aggravating factor in the progression of DN, and inhibiting its activation has also been shown to be effective in delaying the progression of DN (90, 91). The protective effect of aliskiren in DN has also been partially revealed. Mahfoz et al. demonstrated that aliskiren treatment restored blood glucose levels, increased insulin levels, protected kidney functions and relieved renal pathological injury in diabetic rats (92). Similarly, aliskiren could increase insulin levels by increasing glucose transport in the liver and muscles. In addition, aliskiren inhibits the synthesis of profibrotic and proinflammatory cytokines, thus slowing the renal fibrosis of DN (93). Interestingly, the mechanism of renal protection of aliskiren in the DN state is not achieved by lowering blood pressure. After 6 months of follow-up, aliskiren was found to reduce albuminuria levels in DN patients, but did not change the glomerular filtration rate or blood pressure (94). Studies have shown that the inhibition of ER stress may be one of its molecular mechanisms. Aliskiren intervention significantly inhibited the level of ER stress in renal tubule cells induced by palmitic acid (95, 96), and aliskiren combined with chymostatin further inhibited the endoplasmic ER, thus alleviating kidney injury (95). Similar results were also observed in another study. Aliskiren notably inhibited the level of ER stress and simultaneously inhibited the expression of profibrotic growth factors and proinflammatory cytokines, thereby ameliorating the renal injury in DN mice (97). These results suggest that aliskiren may be an effective inhibitor of ER stress.

### 5.4 Valsartan

Valsartan is also a class of angiotensin II receptor antagonists commonly used clinically, and its role in delaying the progression of diabetic nephropathy has also been verified. Moreover, it has also been identified as a potent ER stress inhibitor. It was reported that valsartan could delay the progression of diabetic cardiomyopathy by blocking the activation of the CHOP/Puma signaling pathway to inhibit ER stress (98). Similarly, valsartan can inhibit ER stress induced by autoantibodies against the angiotensin II type 1 receptor (AT1-AA) and thus reduce cell apoptosis (99). In addition, LCZ696 is a 1:1 combination of valsartan and AHU377 (sacubitril), which is synthesized through a complex chemical reaction (100). Belali et al. showed that LCZ696 could effectively restore the mRNA and protein levels of ER stress marker proteins in myocardial cells under diabetic conditions (101). In addition, sacubitril/valsartan intervention can significantly downregulate adriamycin-induced cardiotoxicity in rat models and H9c2 cardiomyocytes, and these protective effects may be achieved by inhibiting ER stress (102). Similarly, LCZ696 can improve chemotherapy-induced testicular atrophy by inhibiting ER stress and apoptosis (103). These results suggest that valsartan may ameliorate renal injury in DN by inhibiting ER stress.

### 5.5 Others

In addition to the drugs mentioned above, some herbals and their extracts have also been found to inhibit ER stress and relieve disease progression. Quercetin is a common flavonoid that is abundant in fruits and vegetables consumed daily (104). Moreover, it is also a strong antioxidant and an ER stress inhibitor. Quercetin can activate the SIRT1/AMPK signaling pathway to inhibit ER stress, thus alleviating osteoarthritis (105) and sepsis-induced acute lung injury (106). Moreover, its glycosylated derivative quercetin 3-O-xyloside also inhibits ER stress by inhibiting the production of ROS in cells (107). In addition, Lycium barbarum polysaccharide, an extract from Lycium barbarum, has also been found to inhibit ER stress and thus improve nerve injury (108) and skin injury (109). Here, we summarize some of the agonists and inhibitors of ER stress (**Table 1**).

**Table 1:** Inhibitors and activators of ER stress.

Compounds	Targets	Clinical trials	Effects on ER stress	Ref
TUDCA	All	Yes	Inhibitor	(110, 111)
4-PBA	All	Yes	Inhibitor	(112)
Omega-3 PUFAs	All	Yes	Inhibitor	(113)
Cerebral dopamine neurotrophic factor (CDNF)	ATF6 IRE1α	Yes	Inhibitor	(114)
Aliskiren	All	Yes	Inhibitor	(95)
Valsartan	All	Yes	Inhibitor	(99)
sc-222227	PERK	No	Inhibitor	(115)
Febuxostat	GRP78	Yes	Inhibitor	(116, 117)
L-F001	All	No	Inhibitor	(118)
Memantine	All	No	Inhibitor	(119)
Trierixin	XBP1	No	Inhibitor	(120)
BMS309403	All	No	Inhibitor	(121)
Oprozomib	PERK, ATF6	Yes	Activator	(122)
Curcumin	XBP-1, IRE1α	Yes	Activator	(123)
Lobaplatin	PERK	Yes	Activator	(124)
Delanzomib	PERK	Yes	Activator	(125)

## 6 Conclusion and perspectives

The ER plays an integral role in protein synthesis, lipid metabolism, and Ca^2+^ homeostasis and abnormal ER homeostasis aggravates the progression of DN. Here, we review the role that the ER plays in cellular life activities, and we also describe the maintenance of ER homeostasis by ER stress and ER-phagy. Subsequently, the roles of ER abnormalities in the pathogenesis of DN were also summarized. Finally, we also summarize some ER stress inhibitors identified thus far. The dysregulation of ER homeostasis is involved in the pathogenesis of DN. There are still many problems that must be solved. What is the mechanism by which ER stress is activated in DN? What role does ER-phagy play in the pathogenesis of DN? Dose mutual regulation occur between ER-phagy and ER stress? Which renal cell in the kidney is most affected by ER stress? These questions need to be answered in the future. Moreover, the discovered ER stress compounds or drugs often have other biological effects while inhibiting ER stress. Therefore, it is urgent to develop new drugs targeting ER stress. In the acute state, the occurrence of ER stress often contributes to the homeostasis of ER, while the persistent ER stress will aggravate the injury. Therefore, how to maintain the balance of ER stress is also a problem that needs to be paid attention to. In summary, the ER is one of the most metabolically active organelles in the cell, and thus, maintaining ER homeostasis is a potential target for the treatment of DN.

## Author contributions

MY the first draft of the manuscript. CBL, NJ, YL, SL, HZ, CRL, YH, WC, LL, LX, provided consultations on the preparation of the work. LS contributed to manuscript revision, read, and approved the submitted version. All authors contributed to the article and approved the submitted version.
